# Diseases of the Larynx

**Published:** 1838

**Authors:** 


### Art. IX.—Diseases of the Larynx.

*Traite pratique de la Phthisie Laryngee, de la Laryngite Chronique, et des Maladies de la Voix.* Par MM. A. Trousseau and H. Bellog.

*A Practical Treatise on Laryngeal Phthisis, Chronic Laryngitis, and Affections of the Voice.* By A. Trousseau and H. Bellog. Ballière, Paris: 1837.*

Such is the title of a work which appears to be a prize essay, accepted by the Royal Academy of Paris; and it does great credit to its authors, who have evinced untiring research, and a profound acquaintance with medicine. M. Trousseau is known as a Professor attached to the Faculty of Medicine of Paris, Doctor of the Hospitals, member of the Legion of Honor, &c.

The diseases to which this work is devoted, are assuming a painful interest in the United States. They are often fatal; and their victims are frequently our most valuable citizens, clergymen, members of the bar, and public teachers. We hail, therefore, with pleasure, the production of Trousseau; hoping that his clear views of pathology, and his experience in the treatment of

*A young medical friend has favored us with the following analytical and abbreviated translation, of this valuable work, which we hope will prove acceptable to our readers. Editors.

these affections, will throw some light upon what have been justly considered obscure maladies, because of the difficulty of diagnosis, and the still greater difficulty that has existed, of administering efficient remedies. The therapeutic agents of M. Trousseau, and their application, appear at the first glance so perfectly simple, and founded upon such plain physiological and pathological facts, observed every day in other organs of the economy, that we only wonder nobody ever presented them before.

The book is divided into seven chapters, in which the history, causes, symptoms, treatment and terminations are fully and systematically presented. These we propose to examine in order, hoping fairly to give an abstract of the author's views, though encountering the difficulty ever attendant on studying them in a foreign language.

Our authors claim the most extended signification for the word *Phthisis;* which they define, "any chronic disease accompanied by hectic fever, and emaciation (*consomption.*)" Under this general head are included, chronic lesion of the kidneys, with suppuration and the consecutive hectic and marasmus; white swellings, Potts' disease of the spine, ulcerating cancerous tumours, &c. &c.; and they appeal, in support of this view, to custom, which has attached to the word Phthisis, specific names, pointing out the peculiar local origin of the series of symptoms in differeut cases.

Consumption being more frequently produced by pulmonary tubercles, than by any other anatomical lesion, some writers confine the term Phthisis to that affection, and apply to it this term, even before the system has suffered marasmus; but in such cases the *consumption* would not occur till the last stage of phthisis. Our authors complain of this use of the word, which they consider a perversion of its true sense, and likely to produce vexatious confusion in medical language. Thus we find it used to express the *presence* of tubercles in the lungs, the *state* of the patient who has them, and even the specific predisposition to them. A question proposed by the Academy, shows the extended signification they attach to the word, and that they apply it to other affections than the pulmonary. We must admit, with them, the necessity of terms of specific designation. One of these prefixes is laryngeal, and hence "laryngeal phthisis," is used to signify "a chronic affection of the larynx, able of itself to produce consumption;" but admitting the possibility of such termination of laryngitis, it appears, from the numerous cases cited, that this is a rare occurrence; because, from the anatomical disposition of the parts, the patient commonly dies from suffocation, before arriving at the stage of true consumption—hectic fever with marasmus. Nevertheless, our authors say of such a death, that it was from *laryngeal phthisis*, and therefore give the more extended definition, "every chronic alteration of the larynx, which may bring on consumption, or death, in *any* way."

HISTORY.

*Chap. I.*—Messrs. Trousseau & Bellog affirm they can find very little information upon this subject, for even modern writers seem not to have had one precise idea respecting it. Hippocrates says not a word. What Galen has written shows his imperfect acquaintance with this lesion; and Aetius, so often cited, has almost literally copied Galen, and agrees with him, that these diseases are *easily* cured. Morgagni first gave detailed accounts of them; his cases, however, do not prove that he understood their true pathology, but supposed his patient would die from suffocation only. It is surprising, that his reflections did not lead him to consider more attentively the ulcers of the larynx of which he speaks, and to conceive the possibility of consumption arising from a succession of them, in the upper part of the air passages. Borsieri stated, that the larynx and trachea might become the seat of ulcerations capable of producing hectic; and he is the first who treated of the disease in its true sense, though he seems to have written under the influence of a preconceived theory, rather than from observation. We are also told, that M. Cayol seemed to comprehend the disease; that before him, in 1806, Messrs. Sauvée & Lagnelet had adverted to alterations of the larynx capable of producing laryngeal phthisis; about the same time, M. Double read, in an interesting paper, before the Medical Society of Paris, his opinions, that laryngeal and tracheal phthisis are one and the same thing, attacking the air passages a little higher or lower. Pravaz is, also, mentioned, with praise, for the care with which he gathered the histories of interesting cures. Above all, particular mention is made of the "precious" observations of Louis, Andral and Bouillaud.

Laennec is not mentioned, nor do these affections appear in his writings; for his mind was occupied with the important affections of the chest, which could be detected by physical signs; but he seems to confine phthisis to the lungs, and from some of his remarks on croup, &c. he may have intended to include under it, what our authors call laryngeal phthisis. So much confusion arises from confounding different diseases under the same name, and by giving different names to the same affections. Mackintosh only separates *laryngitis*, which we understand to be the equivalent of laryngeal phthisis, from croup, by its locality; but admits that croup may involve the larynx as well as the bronchia. Good, in his Study of Medicine, class 3, order 2, gen. 7, species 5, has described under "empressma laryngitis," a disease distinct from croup, and mentions the chronic form. Beck and Morton, of our own country, have described the affection; and the latter agrees with Louis, in supposing it commonly a consequence of tuberculous softening in the lungs.

In *chapter II.* we find the *organic alterations* treated of, with sufficient minuteness—embracing not only the lesions of the larynx, but those of the pharynx and its appendages, and the trachea and bronchia, with their ramifications. The anatomical lesions of the larynx, which we conceive, more especially, to belong to the disease before us, are divided into two great classes affecting the mucous membrane and the cartilages. We are reminded "how fallacious is redness as a sign of previous inflammation, so much insisted on by some pathologists, and of the frequent mistakes which may arise from that fruitful source of error, when we remember, that how bright soever it may have been during life, it may completely disappear after death. A want of due attention to this, has led Bayle and others to consider a pale and tumefied state of the aryteno-epiglottidean ligaments and vocal chords, sufficient proof of œdema, and as evidence against active or chronic inflammation; but our authors consider the enlargement, without redness, of the lips of the glottis, a certain sign of inflammation, and that this enlargement itself, may, in some degree, disappear; whereas, redness is often caused by infiltration to the most depending parts, after death, and is unconnected with disease. "Grave errors have arisen from the neglect of these plain truths, and theories respecting laryngeal affections, otherwise very ingenious, have been based upon the wrong impression of facts. Thus

"spasmodic croup" has been established by nosologists, to account for the death of infants, when autopsy showed no false membrane, nor anomalous redness, nor swelling sufficient to produce suffocation. This division of disease would disappear from our catalogues, (where alone it exists,) if more correct views of the pathology and anatomical lesions were obtained; in the same way, when, in the course of a laryngeal phthisis, suffocation has closed the scene, as the swelling and redness may not always suffice to explain this sad termination, asthma, or some other nervous accident, has been introduced to account for it. Œdema of the mucous membrane of the larynx, a rare disease, has been made to assume an important place; it was sufficient to see the vocal chords, the aryteno-epiglottidean ligaments, tumefied and discolored, without very evident traces of inflammation, to make a new specific disease, "œdema glottidis." This disease is not denied; but tumefaction, even without color, is generally evidence of a phlegmasia.

Ulcerations are divided into erosions and ulcerations proper.

*Erosions* involve only the mucous coat; the ulcerations are seated in the subjacent cellular tissue, or carious cartilage. Our authors agree with Louis, that the pus of phthisical patients may produce ulcerations, and this more commonly in the larynx than the trachea.

*Ulcerations* sometimes invade the whole larynx, the chordæ vocales, the aryteno-epigottidean ligaments, and the mucous membrane of the epiglottis; still deeper, they attack the cartilages, which become necrosed or carious, generally commencing in the mucous membrane, though cases are cited, where they communicated directly with a necrosed cartilaginous surface.

Among the alterations of the cartilages, including the epiglottis, the most interesting is ossification, which is considered a purely physiological phenomenon, the result of age; seen also in laryngitis of two years' standing, even in young patients. The analogy between this and callus, from inflammation, consequent upon fracture of the bones, is well explained; and we are called upon to admit, that "the inflammatory afflux in the neighborhood of the periosteum, or cartilage, raises an action in these tissues, in virtue of which there is an osseous secretion effected, which is a singular phenomenon, as inflammation produces the same results as senile weakness. The same facts applied to the cartilages of the larynx, make us understand how, in laryngeal phthisis, ossification is so common and premature a phenomenon." The ossification, we are told, is developed by irregular plates upon the surface of the cartilage, sometimes extending the whole thickness at certain points, or, if general, the flakes from opposite sides are represented as advancing to meet in the middle. The posterior surface of the cricoid is most easily affected, next the thyroid; the arytenoid have never been seen ossified. Our authors do not deny the possibility of such an occurrence, but suppose it to be rare. One case was met with, in which the perichondrium was ossified; this is analogous to the lesion of the costal cartilages. It is insisted, that ossification may exist without any ulceration or erosion, but simply in a case of chronic laryngitis, and be separated by hypertrophied cellular substance, from the ulcerated surfaces.

Necrosis of the cartilages appears very frequently, having been discovered in more than half the examinations. As this lesion has scarcely been hinted at, by other writers with which we are acquainted, the authors shall speak for themselves:

"The necrosed portion is always completely denuded. This is a permanent pathognomonic sign; for dead parts will ever separate from the living; but this separation takes place in a peculiar manner:—the necrosed portion is never covered with cellular tissue, but always completely denuded on its external face; and reposing upon it, is the pus, either secreted at the point of contact, or the bottom of a fistulous track opening into the larynx. Thus sequestra may be formed, and though the separation rapidly takes place between them and the soft parts endowed with great vitality, it is not the case with the cartilage, but the sequestrum requires a long time for its elimination. The necrosed portion is always ossified, though in fevers of a grave type we sometimes find in the larynx, necrosis of the cartilages, without ossification."

In explanation of this, our authors, thinking that ulceration is mostly, if not always, the cause of necrosis, refer us to the law, *ubi irritatio ibi fluxus*, to account for the osseous effusion into the cartilage subjacent to the inflammation. Then, the ulceration reaching the ossification, the latter becomes necrosed the more readily as it is imbued with less vitality than sound cartilage. This is a very plausible explanation; the more so, as, from an examination of the cases, we find caries, instead of necrosis, when the disease runs its course rapidly; and when it occurs in young children, whose cartilages are yet endowed with great activity; further, we are assured, that the arytenoid ligaments and the epiglottis, have never been found ossified nor necrosed, but always carious when affected. Caries, however, appears less common than necrosis; it has often been observed in the rings of the trachea, never in the cricoid cartilage, once in the thryroid, three times in the arytenoid, once in the epiglottis, in the course of two years. In caries, the ulceration commences in the mucous membrane; increasing rapidly, it extends to the submucous cellular tissue, so as to reach the perichondrium and cartilages, in a few months, or perhaps weeks. This extreme rapidity is explained by the coincidence of tubercular pulmonary phthisis, and by resembling its fatal tendency to ulceration and suppuration. Examining the carious parts before dissection, we are told that the bottom of the ulceration resting upon the cartilage is active, and possessed of considerable vitality; the dissected cartilage is eroded, and appears gnawed. In some instances this erosion has completely destroyed the epiglottis and arytenoid cartilages, and in one case the thyroid was perforated, as exhibited in one of the plates. Andral mentions a similar case, where a perforation at the angle of the plates of the thyroid, established an air passage.

These lesions do not occur singly, but are almost always accompanied by other alterations yet more alarming. Indeed, it is impossible, that deep ulceration, caries or necrosis, should exist without considerable inflammatory engorgement, existing in the mucous membrane and subjacent cellular tissue. This is what has been called *œdema* by Bayle, and becomes the immediate cause of death by suffocation. The name is altogether objected to in the work before us. The œdema glottidis of Bayle, which has given rise to so much discussion among writers, being supported by some of the highest in the profession, and utterly denied by others. Trousseau says, he has established, by nine dissections, two of which were acute cases, developed by a violent catarrh and by bronchotomy, and seven were chronic. Five of these were accompanied by necrosis, caries and ulcerations—two by ulcerating tumours of the larynx.

The remainder of this chapter is occupied with the alterations which, commonly, occur in laryngeal phthisis. Among them, polypi are mentioned; but appear very rare. May they not have been causes of the disease? The case observed by Trousseau is highly interesting, as it exhibits a "tuberculous or polypous" tumour, without any organic pulmonary lesion. Vegetations, syphilitic and cancerous have been observed, but are uncommon. Tuberculous tumors, or granulations, when existing coetaneously with tubercle in the lung, may or may not be ranked with tubercular productions. Louis says, tuberculous granulations are not found in the air passages; whence he concludes, that we should regard these affections as the result of inflammation. Andral (*Clinique medicale*) thinks they are not tuberculous. Our own Morton admits, with Louis, that laryngeal and tracheal ulcerations, are commonly consequent upon tubercle of the lungs, and may be excited by the pus in expectoration; but he acknowledges they may exist independently of any pulmonary affection. Tumours in the neighborhood of the larynx may often simulate laryngeal phthisis, and may cause every symptom of phthisis. In one hundred and two subjects, examined by Louis, eighteen had ulceration of the epiglottis, twenty-three had ulcerations of the larynx, thirty-one lesions of the trachea; from which we see that the frequency of ulcerations increases as we approach the lungs. In the eighteen cases of ulceration in the epiglottis, twelve were in men, six in women; five times they were not complicated with other lesions. They were generally superficial, twice only they reposed on the fibro-cartilage, and, one case excepted, they were on the laryngeal face of the organ; in four cases the circumference was eroded; once it was completely destroyed. The lesions of the larynx, twenty-three times seen in one hundred and two cases, were sixteen in men, seven in women, twice only they were not complicated with either epiglottis or trachea. Of the thirty-one cases of lesion of the trachea, there were twenty-two men and nine women, the site was generally near the bifurcation, the cartilaginous rings were frequently eroded or destroyed; in five cases only, the mucous membrane was destroyed over the whole of the membranous portion of the trachea. Hydatids are mentioned, but must be very rare. False membranes are spoken of, as one of the alterations; but here we are trenching upon another disease—cynanche trachealis, or laryngitis of our writers. Calculi have been observed by Pravaz and Lieutaud. Foreign bodies may be introduced, and cause immediate suffocation, or subsequent phthisis. Though from the commencement of this century, and since Cayol's monograph appeared, most writers have made a distinction between laryngeal and tracheal phthisis, our authors reject the arrangement, and with reason, since no one contends that they do not arise from the same circumstances, under the influence of the same predisposing or exciting causes; and, when sympathetic, the same diseases occasion both. They do not consider the distinction useless, but that it is not sufficiently important, to make two distinct affections, which grow out of one cause, with the same anatomical lesion, and with symptoms and treatment differing so little.

The *third chapter* treats of causes, and is introduced by our authors, as follows:

"Laryngeal phthisis is not a disease *sui generis*, as it is nearly always presented with analogous forms. It may exist alone, and without our perceiving any thing in the economy to explain its development, but is generally a consequence of some organic lesion, which, once established, becomes a true cause."

We will not stop to discuss the correctness of this plan; but it is evident, that the organic lesions alluded to, must be of an extremely varied character; and if we embrace them all under the head of laryngeal phthisis, because they generally provoke nearly similar symptoms and functional disorder, depending rather upon a specific afflux to the organ, than upon their intimate nature, we must review the whole nosological table. Our authors enumerate a great many ways, in which they think the disease has been excited; in some, perhaps, we may be allowed to think, they had no better argument than "*post hoc ergo propter hoc*"—so slight does the connection appear; but they refer principally to phthisis pulmonalis, and, in general, to those constitutions which are most apt to develope chronic ulcerative diseases, especially the scrofulous and tuberculous. In regard to the effects of age upon the susceptibility to this disease, we find the same opinions advanced by most authors:—it is rarely developed before 20, nor after 50 years. From Louis' beautiful work on consumption, it is found that in the tuberculous, at least, there are two men affected to one woman.

In the next chapter, we are informed of the different "species," or, perhaps, it would be more correct to say, varieties under which it may be presented; and, indeed, in reading some of the cases, we could not refrain from making, in our own mind, a very different diagnosis, and merely considering this disease as a manifestation of laryngeal symptoms. There is a great difficulty in classifying diseases, so as to satisfy both the spirit and truth; indeed, the great defects in all attempts that have been made to classify diseases, almost induce the belief, that a correct nosological table would be a superhuman labor. For diseases are complex things, as our authors observe, depending, at the same time, upon the causes which produce them, the constitution of the patient, and so many circumstances that may modify one or the other, at every moment, that what may have been true in one case, will not be so in another, nor even in the same, after any change in surrounding circumstances has taken place; further, "diseases are functions, and not things," and these anormal functions may, like normal ones, vary in every individual, and in every condition of life. Whether we class diseases according to symptoms or anatomical lesions, which are both cause or effect, we shall omit one of the elements of the problem, and have defective classification.

Our authors make four species depending upon their causes:—

1st. Simple laryngeal phthisis—our laryngitis—the empressma laryngitis of Dr. Good. This, though long contended, is no doubt sometimes found unconnected with any other morbid derangement. It is generally believed to have been fatal to our Washington. *M. Double* was the first who proved the separate existence of this form of disease. He says, "without doubt, laryngitis is, in most cases, connected with tubercle of the lungs; but it is in many cases unaccompanied by any such lesion; many cases have, by symptoms, ausculation and autopsy, established this proposition."

2d. Syphilitic laryngeal phthisis, is that produced by venereal ulcers, primitive or consecutive, whether they attacked the larynx in the first place, or were propagated from the pharynx.

3d. Cancerous, from a cancerous tumour in the larynx. These two, depend too much upon accident, to constitute separate species; and might, with great propriety be placed with the preceding or the following, upon the clearer specific distinctions which separate them.

4. Tuberculous laryngeal phthisis, which appears by far the most common form, embraces all those which commence after the manifestation of pulmonary tubercle.

Perhaps, say our authors, there may be another species, the *tetter*, or *ringworm*, (*dartreuse*,) but they do not seem to have made out the case very clearly, and so we leave it.

We now come to a more important department of the work—the symptoms—which are first given as generally appearing in laryngitis; then those presented by the different forms; and some notice taken of the diseases likely to be mistaken for it.

"The local symptom of most value is the alteration of the voice, sometimes a simple weakness, at others a hoarseness, either constant, or only occurring after the larynx becomes fatigued, or the patient is exposed to change of temperature; and it is somewhat singular, that an elevation has most effect, but such seems to be the fact. The hoarseness increases towards evening, and when the patient is hungry; and is diminished for a short time after eating or sleeping. The voice is observed to become hoarser at the period of menstruation. At first intermittent, it becomes constant throughout. The physical signs of hoarseness are difficult to describe, but a practiced ear detects those which answer to certain alterations of the larynx. Some hoarseness will indicate a mucous or broken sound, which proves that the column of air is not free in its passage. This we call the *mucous.* It is heard in simple colds—indicating simple catarrhal affections. In other cases the voice is hoarse, unequal and rough, which we call *strident*, or stridulous. This is a grave symptom, generally answering to ulceration or vegetations."

By aphonia, we mean a complete abolition of the faculty of producing sounds. It comes on suddenly in the second stage of the disease, and is a bad symptom. Its severity is always subordinate to several conditions, which singularly modify it. That aphonia which comes on suddenly in the acute form, and persists in the chronic stage, is not nearly so troublesome, as that which comes on gradually; nor is it so severe after the mucous hoarseness, as after the stridulous."

Several peculiarities in the alterations of the voice are mentioned, all very interesting as philosophically dependent upon the changes effected in the calibre and action of the larynx.

The cough appears to offer nothing particularly instructive, it is generally more frequent, and fitful. The sound of the cough always participates with the voice; it takes a peculiar sound in the stridulous hoarseness, which is called *eructante*, because when the patient coughs, he seems to make a suppressed eructation. This is always a sign of serious alteration of the larynx, and that the glottis is unable to move, or that ulceration prevents its being closed. The difference in frequency of the cough is inexplicable.

With some patients it is incessant, so that they cannot enjoy a moment's repose, and the aliment they attempt to swallow, is ejected by the expiratory muscles; others, whose autopsy may show the same lesions, have scarcely coughed, and have only succumbed under the asphyxia produced by the swelling of the mucous membrane of the larynx.

The expectoration furnishes signs which are rather negative than positive. It is commonly mucous, transparent, little tenacious, sometimes very abundant. When there is ulceration, some new signs are observed, little puriform masses, often mixed with striæ of blood, or quite bloody, are thrown off, rather by hawking (exscréation) than by a cough.

It appears that pain is not a constant attendant upon the disease, being absent in more than half the cases; and it is somewhat remarkable, that those who suffered a little in the onset, when the disease was acute, ceased to suffer when the mucous membrane and the cartilages were entirely destroyed by ulceration and necrosis. With the smaller number there is a little pain in the larynx, and especially at the origin of the trachea; but this does not greatly incommode the patient; on the contrary, nearly all suffer acutely in deglutition, when they do not feel the effort of speaking. This, we are told, is because the mucous membrane investing the epiglottis, the aryteno-epiglottidean ligaments and the arytenoid cartilage, is almost always the seat of considerable inflammatory engorgement; but this part of the larynx constitutes the anterior wall of the pharynx, and no effort at deglutition can be made, without pressing against these inflamed and often ulcerated parts; but when we touch the larynx in front, and through the skin, the inflamed mucous membrane is protected. Add to this, the greater degree of sensibility of the membrane at the upper part of the larynx, than at its interior, as has been observed in tracheotomy.

The signs obtained by sight, enable us to judge it probable, that the larynx is affected when we observe inflammation in the pharynx, combined with other symptoms of laryngeal affection; and the exploration of the epiglottis is of extreme importance, for though it be attached to the tongue, it must be considered pathologically as an appendix to the larynx, and generally partakes of its lesions. How are we to see it? "By the aid of the speculum laryngis;" but there is not one patient in ten who can bear its introduction. The glottis is altogether out of sight with the speculum, even in the dead subject.

Our authors very ingeniously explain the absence of swelling on the anterior part of the neck, (which they have never seen, in simple laryngitis,) by the interposition of the cartilage, between the subcutaneous cellular substance and the inflamed lining membrane of the larynx, and add, that it is remarkable it should so seldom occur, even in cases of considerable necrosis, or caries of the cartilages. In a very interesting case which has fallen under our notice, which we think, in this classification, would rank as a simple laryngitis, now chronic, there is a considerable enlargement around the thyroid cartilage. We can learn very little from touch; the crepitus which some writers have observed, has been found in a sound larynx; touching within the mouth is confined to the epiglottis, and to a second of time, before the convulsive action of the parts prevents our continuance of the exploration.

"The signs furnished by respiration are next in importance to those of the voice. In the first stage, nothing very remarkable is observed, unless under peculiar circumstances, and after violent exercise, when the inspiration is a little braying. As the disease advances, the oppression increases; both from the affection of the lungs, if that exist, and from the narrowing of the larynx; as this goes on, the inspiration becomes hissing, and the expiration long and braying; the patient experiences a sense of imminent suffocation, with great anxiety; the fit subsides, but the orthopnœa continues; then the paroxysms are renewed, with increased strength and frequency, until, at last, the patient dies suffocated. The description of the sufferings endured is harrowing to the feelings; but death is preceded by a calm. The time which elapses between the first fits of orthopnœa and death, is usually from fifteen to twenty days; during the last five days, the fits recur more than once, during twenty-four hours. The course and order of these symptoms are very important, because they offer the only means of deciding the propriety and time for tracheotomy." "Bayle and Thullier have insisted, that inspiration is much more difficult than respiration, when the aryteno-epiglottidean ligaments are affected with œdema. This is explained, by the doubled velocity with which the air traverses the glottis during the inspiration, and it produces a hissing sound in other states of the larynx, not observed in expiration; it does not arise then from the free and œdematous edges of the ligaments being forced into the larynx during respiration. This imagined engulphing of the superior borders of the larynx is not so easy as supposed. Indeed, in inflammatory œdema—and it is nearly all of that character—the consistence of the mucous membrane and submucous tissue, is not flaccid, but, on the contrary, there is nearly always an extreme tension. It is true, we may find these parts in the subject œdematized, and so flaccid, that they tremble, and may obey the impulse of air blown into the mouth; but besides the rarity of this phenomena, may it not be purely cadaveric?"

Autopsy shows us a fact, not explained by the symptoms—the incomplete obliteration of the larynx. Attentive observers know, that the glottis is never entirely shut, and that there still remains a passage for the air; from which our authors conclude, not that there was superadded a spasm of the bronchia to the disease of the larynx, and which was finally the cause of death, but that the swelling of the mucous membrane is, decidedly, diminished after death; and consequently the opening of the glottis may appear larger than it really was. But admitting that the same orifice existed in the glottis during life, it does not follow, that life could be sustained; for if there was not asphyxia in its most rigorous sense, an insufficiency of air would extinguish life in the same manner. During life, the glottis can never be entirely closed. That would induce sudden asphyxia, in the course of one or two minutes, instead of the slow asphyxia we observe in croup and laryngitis. If the glottis, having a capacity represented by four, necessary to healthy respiration, be reduced to a capacity of two, and if it be necessary to the aëration of the blood, that the volume of air should correspond to the normal capacity of the glottis, it is evident, that through one half less space, the lungs can receive but half the quantity of air that is necessary to sanguification. Under these circumstances, it is precisely in the situation of the animal in Bichat's experiment, whose blood flowed in the carotid of a vermilion colour, when the trachea was left open, brown when half shut, and black when entirely closed. When, then, the larynx is half closed, the blood is not sufficiently oxygenated, and asphyxia follows.

"Considering now the influence which the blood exercises on the nervous centres, and those centres back again on respiration, we can understand all the spasmodic phenomena, all the mucous symptoms which may occur, and the intermissions, as belonging to the nerves, without any need of recourse to "spasm of the bronchia," which no one has ever seen. To prove the existence of spasm, it is necessary to find some anatomical element, capable of explaining it. Muscular fibre has not been clearly made out in the bronchia; the fibres resemble muscle, rather in color than in texture, and a functional resemblance is wanting."

In several experiments made by Trousseau, upon the bronchia of animals, immediately after death, stimulating them in every way, he tried to rouse muscular movements, but could never succeed in exciting them, though the heart and muscles of the life of relation continued to offer signs of contractility. This enquiry is pushed to some extent; but we must refer to the book, which contains some admirable disquisitions. But for the sake of any who may entertain doubts—for we have high authority for the muscularity of the bronchia—allow us to introduce a very simple experiment:—laying bare the trachea of a horse, you may cut, tear, or stimulate the surface in any possible manner, and you will not perceive the least muscular contraction.

"In the numerous operations of tracheotomy we have performed, we have never perceived muscular contraction in the fibres of the trachea; and when we have penetrated the bronchia with probes, we have never felt any contraction of them on the whalebone, notwithstanding our sponges were often soaked with very irritating liquids."

The intermissions of the fits not being very clearly explained, by any thing we know of, they are turned over to a *law of our organism;* in which the pain manifests itself at intervals, &c. &c. In the case before us, the nerves may exert an influence, but the phenomenon is not to be set down as a nervous one. In some patients, besides the pain of the mere act of deglutition, there is another serious symptom, and one which appears very difficult to explain, the impossibility of swallowing liquids, or such finely divided food, as cannot be made into a homogeneous alimentary ball. Most authors attribute this to the destruction of the epiglottis, thinking that it does not exactly cover the entrance of the larynx; and that thus the food falls into the air passages. Our authors confess themselves unable to enlighten us, as to the mechanism of this functional difficulty; but think they have proved that, in some patients, the destruction of the epiglottis does not hinder them from swallowing perfectly, while others, with the epiglottis untouched, could not execute any effort at deglutition, without having the food penetrate the larynx. Louis gave the following signs, as indicating ulceration of the epiglottis—"fixed pain in the superior part of the thyroid cartilage, or immediately above it, painful deglutition, ejection of drinks by the nose, the pharynx and tonsils being sound." Trousseau observes, that two of his cases showed, that all these symptoms might exist, without ulceration of the epiglottis, and, he says, all may be absent with the epiglottis destroyed.

The symptoms already presented, as belonging to simple laryngeal phthisis, belong also to the other forms. The sound of the voice, cough, puffing, the mode of deglutition and respiration, are exactly the same, because dependent upon an organic change common to all—inflammation, ulceration and narrowing of the larynx. To these are added some specific symptoms. In the syphilitic form, the pain in deglutition is generally very severe, owing to the ulcerated condition of the fauces so often present. When the syphilitic ulceration is confined to the larynx, it is not manifested by any particular sign, and the disease can only be recognized by the antecedent history of the patient, or by collateral symptoms on the skin or bones, &c. This form of disease is generally an extension from the nares or pharynx, and experience shows, that the affections of the larynx are very analogous to those of the gullet; thus an erythematous syphilis of the fauces, is generally followed by laryngitis, without ulcers; and we may presume, that syphilitic ulcers and necrosis exist, in the larynx, when we observe analogous disease in the nasal fossæ, and when the tonsils or palate have been deeply ulcerated. Tubercular laryngeal phthisis may present, besides the symptoms of the simple form, the characters of phthisis pulmonalis. This species is distinguished by stethescopic signs, by the nature and abundance of the expectoration, and the rapidity of the emaciation. Before forming our prognosis in laryngeal disease, we must make the strictest examination of those signs which reveal tubercles in the lungs, because of the tendency which they have to aggravate slight phlegmasiæ, and bestow incurable characters upon them. The cancerous species does not differ, in symptomatology, from the simple; in the only example reported; there were not even the lancinating pains. The presence of the tumour alone can lead to the diagnosis; and as that must be fully developed, before it is certainly known, we should suppose it ought to have been classed with the simple form, among those caused by foreign growths, within or about the larynx.

There are three diseases which may be mistaken for laryngeal phthisis:—tracheal phthisis, supported by Cayol and others, but which our authors make the same disease, because it has the same symptoms, and co-exists with it; the œdematous laryngeal angina, which, when acute, can only be confounded with *croup*, and, when chronic, the most frequent form in which it is presented, is only a termination of laryngitis, and a closing symptom of it; and, lastly, asthma; not that produced by disease of the heart and lungs, which could not be confounded with laryngitis, but that singular nervous state of the respiratory apparatus, characterized by paroxysms of orthopnœa, followed by a calm in the respiration. The phenomena are very similar as to posture, expression, muscular efforts; but one pathognomonic sign suffices to distinguish them—in asthma the voice is sonorous.

The sixth chapter commences with a very able disquisition upon the terminations of disease, in which is examined the question of the propriety of naming diseases which affect different organs, from the one first affected, as in the malady under consideration.

"Other lesions often follow in its train, which may be more grave than the disease of the air passages; still, if they be secondary, we should say, that the patient died from laryngeal phthisis, making the well established priority of lesions the voucher for the name of the disease. If the lungs be first affected, and afterwards the mesentery and follicles of the intestine become the seat of disorders, more immediately destructive than those of the lungs, we should still call this a pulmonary phthisis; so, if the series of general and local phenomena have begun in the larynx, and afterwards, the laryngitis still progressing, the lungs, the mesentery and intestines present signs of tubercle, we still call this a case of tubercular laryngeal phthisis. And if you choose, it may be also a pulmonary phthisis, a tubercular enteritis, or mesenteric atrophy."

The difference between phthisis pulmonalis and laryngeal phthisis, is shown by the great length of time required for the latter to destroy its victim by consumption, while the former destroys with frightful rapidity. They call on us to admit tubercle as a general fact in our economy, attacking all the organs, tho' chiefly the lungs; and that the simultaneous occurrence of lesions, in different apparatus, in no wise proves a necessary connexion between the organ first affected, and those which subsequently suffer. Upon these grounds, we find they claim as cases of tuberculous laryngeal phthisis, all those where an affection of the larynx has been the primary lesion.

Simple laryngitis may cause death. Our authors have never seen a case produce death by consumption, but their observations led them to believe, that it might do so; and they have often observed chronic diseases of the larynx, causing a fever with hectic characters, and considerable emaciation and debility, and a decided consumption, before death was caused by the suffocation attendant upon the swelling of the mucous membrane of the air passages. This is death from laryngitis, in the strictest sense of the word; the only laryngitis, according to some writers, and a very rare disease.

"In ordinary circumstances, the affection of the larynx, when not far advanced, and when it does not *cause* obstinate cough, fever, nor oppression, does not produce emaciation; but when it follows these phenomena, the patient soon falls into a dangerous state, dyspnœa advances, and death comes on with asphyxia, the most common termination."

How can we call this laryngeal *phthisis*, why not rather laryngitis. Moreover, in many cases which are presented, it is difficult to understand how the symptoms of hectic could be produced by a chronic ulcerating phlegmasia of the larnyx. The surface of the mucous membrane is affected to so small an extent, the suppuration is in general not copious, pain so insignificant and the sympathetic relations of the organ so unimportant, that it requires all the authority of names to establish the existence of simple *laryngeal phthisis*. But the secret is developed; the continuance of the cough fatigues the lungs and expiratory muscles, and causes pervigilium, the pain of deglution is often considerable, and in some there is absolute inability to swallow the least portion of food without suffering convulsive cough and suffocation; in a word, want of sleep and starvation are sufficient to explain the cause of death, and in many cases the hectic too. "Tuberculous pulmonary phthisis," appears to be a frequent termination of *Laryngeal phthisis*. Louis proposes in explanation, that it is caused by the irritation of the pus coming from previous tubercles in the lungs, though it may be more plausible to attribute both diseases to a tuberculous diathesis—admitting that they are simultaneous, and that neither is cause of the other, any more than we attribute the colliquative diarrhœa to the tubercles in the lungs, but to the diathesis which disposes both lungs and intestines to suppuration. This generally first occurs in the lungs, but some rare cases establish that it may commence in the intestines and affect the lungs secondarily. There is a still more intimate connexion between the larynx and the lungs, than between them and any other apparatus in the economy: therefore it may not be the laryngitis that cause phthisis as Borsieri thought, nor phthisis which caused laryngitis as Louis considered probable, so that our authors have concluded to confine themselves simply to the question of priority.

Aphonia, the shrivelling of the larynx (retrecissément) and the oppression consequent upon it, are great obstacles to auscultation. We lose the sound of the voice, that furnishes such valuable diagnosis, nor can we perceive other slight changes because the respiratory murmur is masked by the hissing of the larynx. Our authors arrive at the following conclusions which appear very plausible and philosophical.

1st. That tubercular pulmonary phthisis generally exists first and that the larynx is affected afterwards.

2d. That in the rarer cases the tubercular lesion does begin in the larynx, and the lungs are secondarily affected.

3d. That sometimes laryngeal and pulmonary phthisis advance together.

4th. That in this case the disease may seem to exist exclusively in the larynx because of the predominance of the laryngeal symptoms."

The œdema glottidis of Bayle which has been introduced and adopted by most nosologists since his time, is next examined in its relation to laryngeal phthisis and most cases of it appear either to have been mistaken for an inflammatory engorgement, or a termination of the latter affection. The name is very justly objected to because of its being unphilosophical, the Glottis not being an entity cannot be œdematous, "œdematous laryngeal angina" is proposed as a substitute. The diease does not appear very well established.

"Writers who contend for œdematous laryngeal angina, without inflammation depend upon the pale color observed in the aryteno-epiglottidean ligaments, from infitration of pus or seropurulent matter, but these very words presuppose inflammation—the pale color of the palpebræ after erysipelas, is analogous—and constantly observed, though the inflammation was high during life and the parts red. The same thing is observed in the pale or yellow color of the conjunctiva after chemosis, or in the pale pharynx of those who have died with pharyngeal angina. Bayle attempts to make a diagnosis from the manner of inspiration.

Notwithstanding the alarming nature and characters of laryngeal phthisis, a cure may be effected in the commencement of the attack, before the tissues of the larynx and trachea have become too much altered, but there is no hope of a happy termination if the disease have so far advanced as to debilitate the system, by habitual dyspnœa, cough, abstinence, and marasmus, as proved by the general want of success.

In the last chapter we have directions for treatment, which possess peculiar interest to the physician who has been baffled in all his efforts to reach so obscure and unapproachable a lesion. In the first stages, the remedies suitable for a simple catarrh may avert the progress of the disease, but when the peculiar symptoms advance with increasing intensity we must use more rigorous treatment. Repose of the organ is very properly enjoined, speaking should be avoided as much as possible—venæsection in the arm seems to have given better results than leeches applied near the seat of the affection, which concurs with our own experience, in a very interesting case, we could only observe a trifling melioration, which lasted but a short time, from the application of large numbers of leeches. Next to these modes we are advised to use cut-cups to the nuchæ, but we doubt whether much benefit would arise from this means. We are very philosophically advised against the use of hot poultices to the outside of the neck, so often prescribed by some physicians, as they will be very likely to produce a considerable tendency or afflux of blood to the part, and aggravate the symptoms. Revulsives seem to stand in higher favor, blisters to the front of the neck cause too much pain, but setons are much preferred being less painful and irritating and more easily dressed, the most happy effects were produced by a seton about opposite the cricoid space; still, neither these nor antimonials, nor caustic potassa appear to promise much. Whatever be used must be continued for a long time. The potash is applied on either side the larynx and trachea every week so as to have five or six suppurating cauteries succeeding one another. Our authors say they have still less faith in revulsives at a distance from the organ.

As the pain caused by the congestion of the parts, may in itself give rise to inflammation, narcotics have been used to calm this tendency, and relieve also the cough which is very painful when there is an ulcerated surface, we are recommended to use the extracts of stramonium and belladonna, and the salts of morpheum to the denuded cutis. When simple or ulcerative inflammation becomes chronic and only occupies a small part in the economy we know how difficult it is to control it by the best application of general remedies, and how happily topical treatment generally controls the most obstinate ulcers of the throat, mouth, eyes, &c. By discovering a method of topical application to the larynx, so as successfully to combat diseases of this almost inacessible organ, new prospects open before us, for in our prognosis, that which has been considered incurable may be rendered curable. Here we find new modes of administering medicines, by *respiration*, and some new applications of therapeutic agents attract our attention. The grand difficulty is to introduce medicines without obstructing the respiration.

Fumigations have long been used in these disorders, pure or variously medicated, as with chlorine, iodine, hydrosulphuric acid, &c. with some supposed benefit. Trousseau says they have even used vapors of cinnabar, sulphuric acid, &c. with *various* results. The difficulty with fumigations is the danger of irritating the lungs, as it is impossible to restrict their application to the diseased organs. Liquids are certainly of much easier application, and are more under control of the operator; both irritating and astringent solutions are used, consisting of nitrate of silver, corrosive sublimate, sulphate of copper. The nitrate of silver, so beneficial in external affections, is preferable on account of its rapid action, and harmlessness. We are advised to use a solution of ʒj. of the nitrate to ℥.ij. of distilled water, sometimes ℥. iv. water is used. This is to be applied with a piece of whalebone, bent near the end, to which is attached a small portion of sponge. When the application is to be made, we are to open the mouth, depress and draw forward the tongue with a crooked spoon handle, and introduce the probe until it passes the epiglottis, when it is to be depressed and buried in the upper part of the larynx, in this way a drop or two of the solution is applied directly to the ulcerated surface.

When the pharynx is also affected, as is frequently the case, particularly in the syphilitic species, use a probe of a line and a half diameter, bend it an inch from the end, at an angle 45 deg., fasten on firmly a round piece of sponge, six lines in diameter. This is soaked in the solution, the mouth opened as before and the probe introduced, when it passes the isthmus of the gullet, there is made an effort at deglutition which elevates the pharynx; now bring forward the sponge from the gullet, and by this manœuvre, it is brought over the entrance of the larynx, the epiglottis is lifted up, and the solution is easily pressed out into the larynx, the convulsive cough produced at this juncture favors the introduction, vomiting is also sometimes caused. As may be imagined this procedure is very distressing, though we are assured it is not painful: another method is proposed, which is, injecting with a little syringe like Anel's, having a pipe five inches long and curved at the end, the opening must be at least one-fourth of a line in diameter. We are directed to take up a quarter of its capacity of the solution, then elevating the piston fill the rest with air, so as to effect a shower upon the upper part of the larynx and œsophagus at the same time—This instantly causes a cough and regurgitation which throws out any of the solution that may remain in the œsophagus not combined with the tissues, a few swallows of a gargle of salt water will decompose any that remains in the œsophagus that might be swallowed. Educated with all the terror of cautery in any form, we are much surprised to learn that little pain is experienced, and that the animal sensibility is very obtuse in these organs, which are so well supplied with organic sensibility, causing the cough and convulsive movements.

A close analogy of facts and deductions from them induced our authors, when studying the applications of irritating collyria, so beneficial in certain states of the mucous membranes, to attempt similar medication to the larynx. Finding the introduction of fluids very beneficial, though distressing to the patient, and that the sudden constriction of the glottis prevented the application being made extensively, they endeavored to discover some plan of applying medications to the whole surface of the larynx and upper part of the trachea.

"Areteus had prescribed powdered alum for the malignant angina, in the form of *insufflation*—and it has been found by Bretonneau, in his researches on the subject of *Diptherite*, a most powerful means of arresting the spread of false membranes from the pharynx to the air passages. Areteus used a single reed, Bretonneau a tube with a bulb in its course, and traversed by a gauze partition. Our authors recommend a tube of glass 8 or 10 inches long, with a calibre of two lines; into one end are placed three or four grains of the powder to be used, the other extremity is placed as far back in the mouth as possible, the patient after a deep expiration, closes his lips, and by the brisk action of the diaphragm makes a rapid insufflation. The column of air traversing the tube, hurries along the powder, scattering it over the pharynx and a part kept in suspension by the air, passes into the larynx and trachea. You are advised of the entrance, by fits of coughing which the patient should restrain as much as possible, so as to preserve the medicine in contact with the affected parts. These inspirations are to be repeated several times a day according to the state of the larynx, the nature of the powder, and the manner in which it is borne."

These "dry collyria" are powdered sugar, subnitrate of bismuth, calomel with twelve times its weight of sugar, red precipitate, sulphates of zinc, and copper, with thirty-six proportions of sugar, alum with twice its weight, acetate of lead with seven times its weight, nitrate of silver with seventy-two, thirty-six, or twenty-four times its weight of sugar; which proportions may be varied to suit the case and susceptibility of the patient. Of course these powders should be finely livigated. In the first stages when not severe, the subnitrate of bismuth is used, four grains, four times a day. If this be ineffectual, acetate of lead, alum, sulphate of copper or zinc, and nitrate of silver are substituted; the alum is preferable if there remain but little swelling of the mucous membrane without active inflammation. Nitrate of silver is the favorite remedy with our authors, who recommend it in all cases, not only when there is a simple erythema of the mucous membrane, but also when there are erosions and ulcerations, to be applied two or three times a week, and in some cases every night. Calomel and red precipitate they have only administered in cases of ulceration. These remedies are to be used cautiously at first, and pushed when the patient becomes accustomed to them. Mercury as a *general* treatment has effected cures: its action is little understood, but the result no doubt arises from its powerful property of modifying the general state of the organism. The action of iodine, though no better understood, is more constant in its results; as we see it resolving bony enlargements, &c., we are recommended to try its effects in chronic engorgements of the larynx. It has been twice employed, one case successfully, the other not so. We are called upon to place great reliance in sulphurous mineral waters, particularly those of Bonnes and Cauterets; in these, as well as in pulmonary affections, they have exerted a most happy influence upon the patients to whom they have been prescribed.

In those frequent cases which begin with some pharyngeal affection, the disease seems propagated by continuity, and in such, we are directed to cure the primary evil, in hopes of dissipating the secondary symptoms; thus excision of the prolapsed uvula, tonsils, &c. is said to have cured a commencing laryngeal phthisis, which had been caused by disease of the gullet. Instead of excision, we are recommended to apply the nitrate of silver with a sponge, or a powder of six or eight grains, to a drachm of sugar, with the finger, two or three times a week.

"In the syphilitic form, supposing the laryngeal affection to be merely the extension from the gullet, our remedies, whether they be topical or general, should be directed to the primary alteration. Here the inspiration of calomel and red precipitate are advised, repose of the organs, avoid exposure, cauterize the pharynx and upper part of the larynx, every four days, with nitrate of silver, or a solution of chloride of gold, in nitro-muriatic acid, gargle every day with saturated solution of alum; twice a day insufflate of the following powder, three or four grains:

℞ Protochlor. hydrarg. grs. xj.
Powdered sugar ℥ss. M.

Gargle five minutes afterwards with water. Or the following, in the same dose:

℞ Nitrat. argent. gr. j.
Powdered sugar ʒj. M."

Cancerous and tuberculous phthisis, we are told, are above the reach of art. We can only assuage the severity of the symptoms, and may be called upon to perform tracheotomy for the extension of life; but in these cases it can only prolong the agony. The anatomical relations of the larynx prevent the introduction of tubes, except flexible ones, which are apt to be compressed and obstructed, and become themselves obstacles to respiration; so that tracheotomy is all that is left us, as a last resource. In this matter, the authors appear to have had more experience than falls to the lot of our surgeons. They have performed it seventy-eight times. Of these operations, five were for laryngeal phthisis. They give a minute account of their mode, which does not essentially differ from that usually practiced. They recommend instant torsion of the arteries, and the introduction of the canula before attempting to arrest venous hæmorrhage.

After a careful perusal of this work, replete with interesting cases, and admirable deductions drawn from the results obtained in their management, we rise from our task with a consciousness of benefit received, and more than ever convinced, that persevering and untiring efforts are necessary in the prosecution of our science, in which new facts and new data will continue to be developed, so long as the industry of man may be directed to its pursuit. J. A. W.

